# PB.27. Breast screening with MRI in high-risk women

**DOI:** 10.1186/bcr3712

**Published:** 2014-11-03

**Authors:** D Howarth, J Gilmour, N Sibal, CE Holmes, N Forester, L McLean

**Affiliations:** 1 Breast Screening and Assessment Unit, Newcastle, UK

## Introduction

This study evaluates the use of annual breast MRI surveillance for high-risk women, evaluating the recall rate for further imaging, biopsy and cancer detection rate. We have used breast MRI for surveillance of high-risk women since 2008.

## Methods

High-risk women identified by the Regional Genetic Service between 2008 and 2013 underwent annual breast MRI (1.5 T), in some cases in conjunction with digital mammography. This included those with a genetic predisposition of breast cancer (TP53, BRCA-1, BRCA-2) or equivalent high risk and previous thoracic radiotherapy. Women were offered MRI screening between the ages of 20 and 50.

## Results

Over 6 years, 184 women underwent 477 screening MRIs. There were 44 lesions recalled for second-look ultrasound in 39 women. Twenty-seven lesions had a core biopsy, from which 10 malignant lesions were identified. Nine of these were invasive ductal carcinoma, of which all were either grade 2 or 3, varying in size from 4 to 34 mm. One case of ductal carcinoma *in situ *was identified. The remaining 17 lesions were benign. Women who had a normal ultrasound either had an interval MRI after 6 months or were returned to routine yearly screen.

## Conclusions

MRI screening of high-risk women is effective, with a cancer detection rate of 2.1% and a recall rate of 9.2% which are within the standards set by UK national guidelines. There has been one false negative screen to date. Breast MRI screening provides a viable alternative to prophylactic mastectomy for women at high risk of breast cancer.

